# Guideline for the assessment and management of gastrointestinal symptoms following colorectal surgery—A UEG/ESCP/EAES/ESPCG/ESPEN/ESNM/ESSO collaboration. Part II—Good practice guidance | sequelae to benign diseases

**DOI:** 10.1002/ueg2.12659

**Published:** 2024-09-14

**Authors:** Anke H. C. Gielen, Guideline Development Group, Stavros A. Antoniou, Geerard L. Beets, Stephanie O. Breukink, Suzanne Dore, Asbjørn M. Drewes, Hannah Garside, Marc A. Gladman, Deena Harji, Goran Hauser, Therese Juul, Daniel Keszthelyi, Jos Kleijnen, Christos Kontovounisios, Laura Lorenzon, Lisa Massey, Jarno Melenhorst, Helen M. Mohan, Jean Muris, Coco Smit, Yvonne Tillotson, Arved Weimann, Marco Zelic

**Affiliations:** ^1^ Department of Surgery Maastricht University (Maastricht University, including Maastricht UMC+) Maastricht The Netherlands; ^2^ School of Nutrition and Translational Research in Metabolism (NUTRIM) Maastricht University Maastricht The Netherlands

**Keywords:** benign, clinical guidelines, colorectal surgery, gastrointestinal symptoms

## Abstract

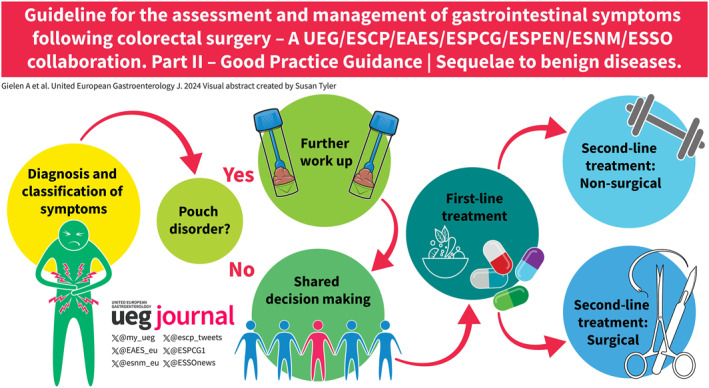

## INTRODUCTION

### Background

Benign colorectal resections are performed to address a spectrum of non‐oncological conditions, including but not limited to diverticulitis, inflammatory bowel disease (IBD), polyposis coli, functional bowel disorders, and endometriosis. While the surgical procedure mirrors that of oncological cases, the primary distinction lies on the preservation of a greater portion of the mesentery in these non‐oncological resections.^1^ Nevertheless, irrespective of the underlying condition, any type of colorectal resection could have an adverse impact on the patient's bowel function.^2^, ^3^, ^4^, ^5^, ^6^

Gastrointestinal dysfunction is a prevalent long‐term complication after non‐oncological colorectal resections. This is particularly the case in diverticulosis patients, where poor functional outcomes have been reported in up to 25% of patients after left hemicolectomy.^3^, ^7^ Bowel dysfunction can manifest with a variety of symptoms, including urgency, constipation, faecal incontinence and/or abdominal pain, all of which require different management strategies.^8^, ^9^ Recent studies have shown that 80% of patients experience late residual symptoms after colorectal surgery, with 70% of these reporting an improvement in symptom profiles following treatment.^10^ Similar positive outcomes were observed in a nurse‐led clinic,^11^ highlighting the clinical and socio‐economic value of recognising and addressing these complications. Urinary incontinence and sexual dysfunction represent additional potential long‐term consequences of colorectal surgery. This guideline focusses primarily on addressing the gastrointestinal symptoms following non‐oncological colorectal resection. All long‐term sequelae can have a significant impact on patients' overall well‐being and quality of life (QoL). For clarity, we will adhere to the term ‘gastrointestinal symptoms’ in this guideline.

Gastrointestinal symptoms can lead to a range of long‐term sequelae following non‐oncological colorectal resections. Each pattern depends on the specific resection type performed due to the differing underlying pathophysiological mechanisms responsible for gastrointestinal dysfunctions.

It is important to note that benign colorectal conditions themselves often involve functional disorders, such as functional constipation, faecal incontinence, or abdominal pain, prior to any surgical resection. These functional aspects contribute to a complex interplay of symptoms.^12^, ^13^, ^14^ Additionally, the neurochemical changes in the innervation of colonic blood vessels in patients with inflammatory bowel diseases (IBD) may contribute to abdominal pain and the altered bowel habit that may accompany this disease.^15^, ^16^

Right sided colonic resections often reduce the capacity for biliary acid absorption.^8^, ^17^, ^18^ Due to the resection of the ileocaecal valve in right sided resections, small bowel bacterial overgrowth may further contribute to bowel dysfunctions.^17^ These dysfunctions may manifest in symptoms such as loose stool, increased bowel frequency, and/or increased nocturnal defecation.^9^, ^17^ Some of these symptoms may improve or resolve spontaneously over time. However, many patients experience persistent bowel dysfunction. We omitted appendectomies if this was the sole resection performed.

Left sided colectomies may lead to symptoms such as diarrhoea, stool fragmentation, a feeling of obstruction and prolonged evacuation time.^17^, ^19^ The primary aetiology is believed to be the reduced capacity of water absorption after left‐sided colonic resections.^8^ Furthermore, the absence of the rectosigmoid junction, which acts as a high‐pressure barrier preventing rapid stool transit into the rectum, may contribute to the development of faecal incontinence.^9^, ^20^ Studies investigating functional outcomes after (oncological) rectal resections have identified the Low Anterior Resection Syndrome (LARS), which is considered to be a condition with a multifactorial aetiology.^21^, ^22^ Key contributing factors include the loss of reservoir function, decreased anal sphincter function, autonomic denervation and afferent sensory loss after rectal resections.^23^ This results in symptoms such as diarrhoea, increased frequency and urgency.^24^ Similar symptoms may occur after resections for benign disorders, although these are not as well described.

Ileal pouch‐anal anastomosis (IPAA) is performed for patients following resection for refractory IBD, who desire restoration of gastrointestinal continuity.^25^ Bowel dysfunction following IPAA is a common long‐term complication, with reported pouch failure rates ranging from 3% to 15%.^26^ Uncontrolled faecal incontinence is a potential contributor to pouch failure.^25^ Defecatory disorders observed in IPAA patients often result from paradoxical contraction and/or impaired relaxation of the pelvic floor and anal muscles during defecation, clinically referred to as ‘dyssynergic defecation’.^27^ For further elaboration on the pathophysiological mechanisms leading to bowel dysfunction, please see ‘Part I—Sequelae to oncological diseases’, of these guidelines.

We are aware that preventive measures are of vital importance in addressing these gastrointestinal symptoms, including considerations of pre‐treatment options. However, the aim of this guideline is to consolidate current evidence on the appropriate assessment and management of gastrointestinal symptoms following non‐oncological colorectal resections.

To our knowledge, no previous guidelines have been published on the assessment and management of long‐term sequelae after colorectal resections for benign indications. Therefore, the aim of this project was to develop an up‐to‐date joint European, multidisciplinary guideline on this topic, using the best available evidence.

### Methods

This guideline has been created in a collaboration with patients and members of the United European Gastroenterology (UEG), European Society of Coloproctology (ESCP), European Association of Endoscopic Surgery (EAES), European Society for Primary Care Gastroenterology (ESPCG), European Society for Clinical Nutrition and Metabolism (ESPEN), European Society of Neurogastroenterology and Motility (ESNM), and the European Society of Surgical Oncology (ESSO). The patient representatives involved in this guideline were selected from the target population for whom this guideline was intended that is, they were patients who currently, or had previously, experienced gastrointestinal symptoms after colorectal surgery. This guideline provides guidance on the relative value of diagnostic modalities and the effectiveness of treatment options for gastrointestinal symptoms following colorectal surgery. The guideline consists of two parts: Part I—Sequelae to oncological diseases and Part II—Sequelae to benign diseases. Both parts contain the following chapters:

‐ Diagnosis
‐ First‐line treatment
‐ Second‐line therapies | Non‐surgical interventions
‐ Second‐line therapies | Surgical interventions

**FIGURE 1:**
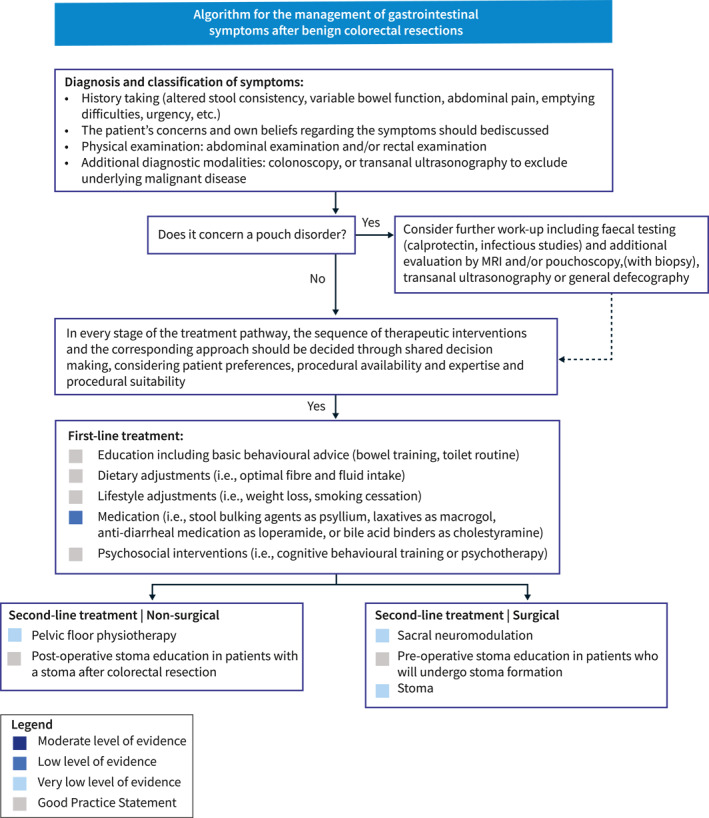
Treatment algorithm gastrointestinal symptoms.

These guidelines are intended for use by all healthcare professionals who treat patients who experience gastrointestinal symptoms after colorectal surgery (e.g., nurses, general practitioners, dietitians, gastroenterologists, colorectal surgeons, etc.). It can also serve as a source of information for patients seeking knowledge about the diagnosis and treatment options for their gastrointestinal symptoms in order to improve QoL. This guideline project was funded by the ESCP and UEG. The Guideline Development Group (GDG) had full control over the development of the protocol and the guideline, without external influence from the funding body. The detailed methods are provided in Supporting Information S1: Appendix I. The evidence‐to‐decision frameworks are provided in Supporting Information S2: Appendix II. Before presenting the systematic literature review, we provide an overview of the recommendations, including a schematic representation in a treatment algorithm (Figure 1).

## RECOMMENDATIONS

**Legend**: Wording and colour of recommendations

**Table:** 

	Quality of the evidence according to GRADE^28^, ^29^	Wording recommendation
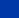	Moderate level	‘Should be used’
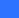	Low level	‘Could be used’
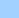	Very low level	‘Can be considered’
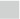	Good practice statement	

**Diagnosis and classification of gastro‐intestinal symptoms after non‐oncological colorectal resections**

**Table:** 

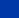	Health care professionals should assess post‐operative symptoms, including altered stool consistency, variable or unpredictable bowel function, abdominal pain, emptying difficulties, involuntary loss of stool or gas and/or urgency.
	*Low level of evidence*; *upgraded by the GDG* (*see evidence to decision framework in* Supporting Information S2: Appendix II)
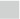	Physical examination should be performed in patients, including an abdominal examination and (digital) anorectal examination.
	*Good Practice Statement*, *ungraded*
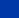	A colonoscopy could be used to rule out anatomical causes (e.g., anastomotic stenosis, pouch‐related complications) for gastro‐intestinal symptoms.
	*Very low level of evidence*; *upgraded by the GDG* (*see evidence to decision framework in* Supporting Information S2: Appendix I II)
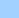	Transanal ultrasonography can be considered as an additional diagnostic modality.
	*Very low level of evidence*
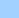	Anorectal or anopouch manometry alone should not be used as a diagnostic modality.
	*Very low level of evidence*
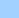	Health care professionals can consider several investigations including a detailed patient history, physical examination including (digital) anorectal exam, faecal testing and additional evaluation by MRI and/or pouchoscopy with biopsy, transanal ultrasonography or general defecography in diagnosing pouch disorders.
	*Very low level of evidence*

**First‐line treatment of gastro‐intestinal symptoms after non‐oncological colorectal resections**

**Table:** 

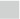	Basic behavioural advice (i.e., toilet routine, bowel training) can be considered.
	*Good Practice Statement*, *ungraded*
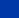	Probiotics should not be used as first‐line treatment for pouch dysfunction.
	*Moderate level of evidence*
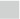	Dietary adjustments (i.e., optimal fibre and fluid intake) can be considered.
	*Good Practice Statement*, *ungraded*
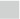	Lifestyle adjustments, including weight loss in overweight patients and smoking cessation in smokers can be considered.
	*Good Practice Statement*, *ungraded*
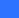	Octreotide or calcium polycarbophil should not be used as treatment for pouch dysfunction.
	*Low level of evidence*
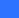	Medication (i.e., stool bulking agents as psyllium, laxatives as macrogol, anti‐diarrhoeal medication such as loperamide, and/or bile acid binders such as cholestyramine) could be used for patients with gastro‐intestinal symptoms or in pouch dysfunction.
	*Low level of evidence*
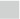	Psychosocial interventions (i.e., cognitive behavioural training or psychotherapy) can be considered once other pathology has been ruled out.
	*Good Practice Statement*, *ungraded*

**Second‐line treatment of gastro‐intestinal symptoms after non‐oncological resections | non‐surgical**

**Table:** 

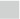	Transanal irrigation should not be used as treatment in patients with inflammatory bowel disease or other anorectal inflammatory pathology.
	*Good Practice Statement*, *ungraded*
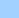	Pelvic floor physiotherapy with biofeedback can be considered for patients with an ileo‐anal pouch, if an experienced therapist is available to guide the patient.
	*Very low level of evidence*
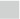	Post‐operative stoma education can be considered in patients with a stoma after colorectal resections.
	*Good Practice Statement*, *ungraded*

**Second‐line treatment of gastro‐intestinal symptoms after non‐oncological resections | surgical**

**Table:** 

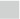	Surgical interventions can be considered for an individual patient if conservative first and/or second‐line treatments have failed to reach sufficient improvement of gastro‐intestinal symptoms after non‐oncological colorectal resections.
	*Good Practice Statement*, *ungraded*
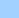	Sacral neuromodulation can be considered as surgical treatment for faecal incontinence.
	*Very low level of evidence*
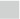	A stoma can be considered in patients with faecal incontinence after non‐oncological (colo)rectal resections for those patients who have refractory symptoms.
	*Good Practice Statement, ungraded*
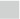	Pre‐operative stoma education can be considered in patients who will undergo stoma formation.
	*Good Practice Statement, ungraded*

## DIAGNOSIS

### Introduction

It is essential during the follow‐up after colorectal surgery, that persistent or post‐surgery gastrointestinal symptoms are appropriately addressed. In patients suffering from benign conditions, the health care provider must carefully determine the exact nature of the patient's symptomatology. A detailed patient history must be taken; several diagnostic modalities can be considered to aid in a thorough evaluation of the symptoms, partly determined by the specific type of resection.

### History taking

A single cohort study by Lovegrove et al. from 2010,^30^ was identified, which examined the essential domains in a detailed patient history concerning pouch function. This study consisted of 4013 patients who had undergone primary restorative proctocolectomy for IBD or familial adenomatous polyposis. To assess QoL, the researchers applied the Cleveland Global QoL score. The symptom domains include stool frequency, urgency, faecal incontinence and medication use (i.e., antidiarrhoeal medication or antibiotics) were all found to be independently associated with the Cleveland Global QoL score. For further details on the relevant items in a detailed patient history, see ‘Part I—Sequelae to oncological diseases’, of these guidelines.

### Physical examination

No studies were identified regarding the role of physical examination in the assessment of gastrointestinal symptoms after non‐oncological colorectal resections. For further details on the role of physical examination, please see ‘Part I—Sequelae to oncological diseases’ of these guidelines. Due to the critical significance of a thorough physical examination in the diagnosis of any medical condition, the GDG has opted to upgrade the level of evidence for this recommendation.

### Diagnostic modalities

For further details on the role of different diagnostic modalities in the assessment of gastrointestinal symptoms after colorectal resections, see ‘Part I—Sequelae to oncological diseases’, of these guidelines. We identified two systematic reviews reporting on the different diagnostic modalities for specifically evaluating post‐operative pouch function.^31^, ^32^ The review by Luo et al. from 2022^31^ included 6 studies, which amalgamated data on 179 individual patients affected by pouch dysfunction and/or pouchitis following IPAA. Overall, less than 10% of symptomatic patients post‐IPAA were referred for anopouch manometry. The prevalence of dyssynergic defecation, as per the Rome IV criteria, ranged from 47% to 100% in symptomatic patients. Faecal incontinence was characterised by decreased mean and maximal resting anal pressure on manometry, as well as patient‐reported hyposensitivity. Additionally, the recto‐anal inhibitory reflex was predominantly absent among patients, in both those experiencing incontinence and those without it. Considering these findings, the authors of this systematic review conclude that relying solely on manometry for assessing pouch function in patients with faecal incontinence is suboptimal. However, it can play a role as one of several diagnostic tools, with further confirmatory testing required, potentially using, dynamic imaging techniques such as pouch defecography.

The review by Roussel et al. from 2022^32^ synthesised findings of 10 clinical studies investigating the management of various functional disorders in patients with IBD who had previously undergone colectomy with IPAA. They presented a range of potential differential diagnoses (i.e., pouchitis, stricture, anastomotic leak, dyssynergic defecation, megapouch, prolapse, infectious diarrhoea, etc.) along with suggested diagnostic modalities to facilitate the thorough assessment of these suspected pouch disorders. As per their recommendations, the initial evaluation should encompass a physical exam including (digital) anorectal examination, microbiological stool tests (e.g., Giardia, Cryptosporidium, Clostridoides), measurement of faecal calprotectin, serum laboratory tests including a complete blood count and C‐reactive protein along with cross‐sectional imaging by contrast‐enhanced CT or MRI and/or pouchoscopy with a biopsy. For specific cases, where dyssynergic defecation is suspected or if rectal examination reveals levator ani tenderness, anopouch manometry may be considered. The authors emphasise the importance of referring patients with refractory pouch problems to specialised centres with expertise in pouch management.

**Recommendations for the diagnosis and classification of gastrointestinal symptoms after non‐oncological colorectal resections**

**Table:** 

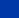	Health‐care professionals should assess post‐operative symptoms, including altered stool consistency, variable or unpredictable bowel function, abdominal pain, emptying difficulties, involuntary loss of stool or gas and/or urgency.
	*Low level of evidence; upgraded by the GDG* (*see evidence to decision framework in* Supporting Information S2: Appendix II)
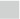	Physical examination should be performed in patients, including an abdominal examination and (digital) anorectal examination.
	*Good Practice Statement, ungraded*
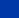	A colonoscopy could be used to rule out anatomical causes (e.g., anastomotic stenosis, pouch‐related complications) for gastro‐intestinal symptoms.
	*Very low level of evidence; upgraded by the GDG (see evidence to decision framework in* Supporting Information S2: Appendix II)
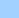	Transanal ultrasonography can be considered as an additional diagnostic modality.
	*Very low level of evidence*
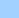	Anorectal or anopouch manometry alone should not be used as a diagnostic modality.
	*Very low level of evidence*
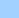	Health care professionals can consider several investigations including a detailed patient history, physical examination including (digital) anorectal exam, faecal testing and additional evaluation by MRI and/or pouchoscopy with biopsy, transanal ultrasonography or general defecography in diagnosing pouch disorders.
	*Very low level of evidence*

## FIRST‐LINE TREATMENT FOR GASTROINTESTINAL SYMPTOMS

### Introduction

Once the patient's concerns have been appropriately identified, the initial steps in managing these symptoms are consolidated as ‘first‐line treatment’. These first‐line treatment strategies aim to alleviate gastrointestinal symptoms following non‐oncological colorectal resections, with the objective to improve QoL. First‐line treatment options include behavioural advice, dietary and lifestyle adjustments, several types of medication (i.e., stool bulking agents or anti‐diarrhoeal medication), and/or psychosocial interventions.

### Behavioural advice

No studies were identified regarding the role of behavioural advice as a first‐line treatment for gastrointestinal symptoms after non‐oncological colorectal resections. For further detail on the role of behavioural advice, see ‘Part I—Sequelae to oncological diseases’, of these guidelines.

### Dietary adjustments

In an RCT by Bengtsson et al. from 2016,^33^ the effect of probiotics on ileal pouch function was investigated. The study enroled 32 patients with ulcerative colitis, of which 16 were assigned to the probiotics group and 16 to the placebo group. These patients had all undergone resection or ileostomy closure more than 1 year prior and experienced chronic pouch dysfunction or a recent deterioration in function. The probiotic treatment consisted of *Lactobacillus plantarum* 299 and bifidobacterium infantis cure 21 diluted in water, and were taken twice daily for a period of 21 days. Pouch function was assessed by use of a pouch function score developed in their own hospital. No difference was observed in pouch functional score or pouchitis disease activity index between the intervention and the placebo control group at 21 days. For further detail on the role of dietary adjustments in treatment of gastrointestinal symptoms after colorectal resections, see ‘Part I—Sequelae to oncological diseases’, of these guidelines.

### Lifestyle adjustments

No studies were identified regarding the role of lifestyle adjustments as a first‐line treatment for gastrointestinal symptoms after non‐oncological colorectal resections. For further details on the role of lifestyle adjustments, see ‘Part I—Sequelae to oncological diseases’, of these guidelines.

### Medication

In an RCT conducted by van Assche et al. from 2012,^34^ the impact of octreotide on pouch function was investigated. The RCT comprised 15 patients with ulcerative colitis, with 9 allocated to the intervention group and six to the placebo group. All participating patients had undergone IPAA and were at least 6 months following ileostomy closure. All patients reported pouch dysfunction based on increased stool frequency, with a median of 9 bowel movements per day. After 7 days of intervention, the median stool frequency exhibited no significant changes compared to baseline values. Additionally, abdominal pain scores were similar in both groups. All nine patients treated with octreotide experienced at least one adverse event, varying from fatigue, worsening of abdominal pain, nausea, constipation, urgency complaints or anal pain. These adverse effects, combined with the scarcity of robust data, led to the GDG to advice against the use of octreotide as first‐line treatment for gastrointestinal symptoms after non‐oncological colorectal resections.

In an RCT by Shibata et al. from 2007^35^ the effect of calcium polycarbophil on pouch function was investigated. The trial involved 16 patients diagnosed with ulcerative colitis who had undergone resection with IPAA and were at least one month following ileostomy closure. Evaluation of pouch dysfunction consisted of anal manometry and a questionnaire on stool frequency, consistency and nocturnal soiling. Patients in the intervention group were administered bifidobacterium (3 g/day) with calcium polycarbophil (3 g/day) for 6 months, while the control group received the same dosage of bifidobacterium alone. Anal manometry measurements before and after the intervention showed no significant differences. Daily stool frequency and night‐time soiling exhibited improvements in both groups, with no significant differences observed between the two groups at the 3‐ and 6‐month follow‐up assessments. Due to the paucity of robust evidence on this topic, the GDG advised against using calcium polycarbophil as first‐line treatment for gastrointestinal symptoms after non‐oncological colorectal resections.

For further details on the role of various types of medication in the treatment of gastrointestinal symptoms after colorectal resections, please refer to ‘Part I—Sequelae to oncological diseases’, of these guidelines.

### Psychosocial interventions

No studies were identified regarding the role of psychosocial interventions, that is, cognitive behavioural training or psychotherapy, as a first‐line treatment for gastrointestinal symptoms after non‐oncological colorectal resections. For further detail on the role of psychosocial interventions, please refer to ‘Part I—Sequelae to oncological diseases’, of these guidelines.

**First‐line treatment of gastrointestinal symptoms after non‐oncological colorectal resections**

**Table:** 

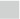	Basic behavioural advice (i.e., toilet routine, bowel training) can be considered.
	*Good Practice Statement*, *ungraded*
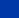	Probiotics should not be used as first‐line treatment for pouch dysfunction.
	*Moderate level of evidence*
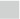	Dietary adjustments (i.e., optimal fibre and fluid intake) can be considered.
	*Good Practice Statement, ungraded*
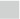	Lifestyle adjustments, including weight loss in overweight patients and smoking cessation in patients who smoke can be considered.
	*Good Practice Statement, ungraded*
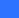	Octreotide or calcium polycarbophil should not be used as treatment for pouch dysfunction.
	*Low level of evidence*
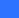	Medication (i.e., stool bulking agents as psyllium, laxatives as macrogol, anti‐diarrhoeal medication such as loperamide, and/or bile acid binders such as cholestyramine) could be used for patients with gastro‐intestinal symptoms or in pouch dysfunction.
	*Low level of evidence*
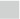	Psychosocial interventions (i.e., cognitive behavioural training or psychotherapy) can be considered once other pathology has been ruled out.
	*Good Practice Statement, ungraded*

## SECOND‐LINE THERAPIES: NON‐SURGICAL INTERVENTIONS FOR GASTROINTESTINAL SYMPTOMS

### Introduction

For patients whose symptoms persist despite initial first‐line treatments, the exploration of additional therapeutic approaches should be considered. In these cases, healthcare providers should prioritise the pursuit of less invasive interventions, that is, non‐surgical interventions, before considering the more invasive surgical alternatives. Treatments should be considered and implemented based patient and physician preferences, and the availability of or expertise in specific treatment modalities. Bypassing non‐surgical second‐line treatment options and opting directly for surgical interventions following first‐line treatment options may be reasonable.

In this section, we will explore a range of non‐surgical second‐line interventions that have demonstrated efficacy in managing gastrointestinal symptoms after benign colorectal resections. By exploring these options, healthcare providers can expand their toolkit for symptom management, tailoring treatments to individual patient needs and preferences. This chapter addresses second‐line non‐surgical treatment options as irrigation methods and pelvic floor physiotherapy for gastrointestinal symptoms after non‐oncological colorectal resections.

### Irrigation methods

No studies were identified regarding the role of transanal or stoma irrigation as a second‐line treatment for gastrointestinal symptoms after non‐oncological colorectal resections. It is thought that transanal irrigation may have an adverse effect when applied in patients with IBD or other conditions affecting anorectal inflammation. Given the potential harmful effects, combined with scarce available evidence and extremely limited expertise worldwide, the GDG recommended against the use of irrigation in patients suffering from IBD or any other conditions affecting anorectal inflammation.

### Pelvic floor physiotherapy

A single cohort study was identified on the impact of pelvic floor physiotherapy as a second‐line non‐surgical treatment for gastrointestinal symptoms after non‐oncological colorectal resection.^36^ The study by Segal et al. reports on 26 patients with ileoanal pouch related problems, predominantly faecal incontinence, evacuation disorders, pruritus ani and/or abdominal pain. All patients were offered six individual sessions with an experienced pelvic floor physiotherapist over the course of 6–8 months. The authors analysed subjective markers of improvement of the symptoms, classified as ‘no’, ‘some’ and ‘much’ improvement. Of the 26 patients, 6 showed ‘no’ improvement after treatment, 10 showed ‘some’ improvement and 10 patients showed ‘much’ improvement. The most pronounced improvement was observed in the groups of patients predominantly suffering from faecal incontinence and evacuation disorders. Despite these results, it is important to acknowledge the limitations of this study. Firstly, the cohort is notably small, the retrospective design warrants caution in drawing definitive conclusions, and that the results are only generalisable to patients with a pouch and therefore, these findings cannot be extrapolated to the broader patient population undergoing non‐oncological colorectal resections without pouch formation. To establish a more comprehensive understanding of the role of pelvic floor physiotherapy, more data is required through robust research studies. We recommend that when an experienced therapist is available to guide the patients through the treatment process, pelvic floor physiotherapy can be considered as a second‐line non‐surgical treatment for patients with gastrointestinal symptoms after non‐oncological colorectal resections.

### Percutaneous posterior tibial nerve stimulation (PPTNS)

No studies were identified regarding the role of PPTNS as a second‐line treatment for gastrointestinal symptoms after non‐oncological colorectal resections. Given the limited available evidence and expertise worldwide, the GDG has opted not to make a specific clinical recommendation on the use of PPTNS in this particular patient population. For further detail on the role of PPTNS, see ‘Part I—Sequelae to oncological diseases’, of these guidelines.

### Stoma education

No studies were identified regarding the role of stoma education as a second‐line treatment for gastrointestinal symptoms in patients with a stoma after non‐oncological colorectal resections. For further detail on the role of stoma education, see ‘Part I—Sequelae to oncological diseases’, of these guidelines.

**Recommendations for the second‐line treatment of gastrointestinal symptoms after non‐oncological colorectal resections | Non‐surgical interventions**

**Table:** 

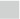	Transanal irrigation should not be used as treatment in patients with inflammatory bowel disease or other anorectal inflammatory pathology.
	*Good Practice Statement, ungraded*
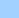	Pelvic floor physiotherapy with biofeedback can be considered for patients with an ileo‐anal pouch, if an experienced therapist is available to guide the patient.
	*Very low level of evidence*
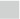	Post‐operative stoma education can be considered in patients with a stoma after colorectal resections.
	*Good Practice Statement, ungraded*

## SECOND‐LINE THERAPIES: SURGICAL INTERVENTIONS FOR GASTROINTESTINAL SYMPTOMS

### Introduction

If gastrointestinal symptoms persist following non‐oncological colorectal resections, despite first and/or second‐line non‐surgical interventions, surgical interventions should be considered. While surgical interventions can alleviate refractory symptoms, they also carry inherent risks. These include direct surgical complications such as infection, bleeding or damage to surrounding organs or tissues, as well as anaesthesiological risks, particularly for patients with underlying heart or respiratory conditions. Additionally, there may be further long‐term sequelae, including altered gastrointestinal function or the need for additional surgeries in the future. Therefore, surgical interventions should always be tailored to the patient and considered on an individual patient basis. This chapter elaborates on the type and timing of surgical interventions as second‐line treatment for gastrointestinal symptoms after oncological colorectal resections.

### Timing of surgical interventions

No studies were identified regarding the timing of surgical interventions as second‐line treatment for gastrointestinal symptoms after non‐oncological colorectal resections. Any indication for surgery and its timing must be individually tailored including a second opinion and should follow the principles of shared decision making. It is essential that the patient has been appropriately counselled and understands the potential risks of complications, prior to deciding for the implementation of surgical interventions.

### Sacral neuromodulation

Two cohort studies assessed the impact of SNM as a second‐line surgical treatment for gastrointestinal symptoms after non‐oncological colorectal resections.^37^, ^38^ Mizrahi et al. in 2017^37^ matched 12 patients with faecal incontinence after a proctectomy (comprising 6 patients with rectal cancer, 6 with benign indications) with an equal number of patients who experienced faecal incontinence but had no history of proctectomy. All 24 patients underwent SNM with The Cleveland Clinic Florida Faecal Incontinence Score (CCF‐FIS) used to monitor the severity of the experienced symptoms.^39^ Within‐group analyses suggested a significant improvement in pre‐and post‐operative median CCF‐FIS of patients in the SNM after proctectomy group. Interestingly, this improvement was more pronounced among patients who had previously undergone a proctectomy for benign indications as opposed to those who underwent the procedure for rectal cancer.

The retrospective cohort study by Seifarth et al.^38^ reported 23 consecutive patients who received SNM for increased stool frequency or faecal incontinence after proctocolectomy with IPAA for ulcerative colitis between 1993 and 2020. The median follow‐up time was 6.5 years, two patients were lost to follow‐up. Median time from ileostomy closure to SNM implantation was 6 years. This study used the St. Marks score for anal incontinence to monitor the patients' symptoms.^40^ The score improved in 16 out of 23 patients after SNM patients, with a median change from 19 to 4 points. SNM treatment was ineffective in 7 patients. After a control period of at least 3 months, the electrodes were removed. Reasons for failure of the treatment and removal of the electrodes were severe pouchitis in three patients, persistent incontinence or severe anal pain. The authors conclude that SNM implantation could be a feasible treatment option for this particular patient population, and that awareness of the possible beneficial effects of SNM should be increased in healthcare providers treating these patients.

### Stoma

No studies were identified regarding the role of stoma formation as a second‐line surgical treatment for gastrointestinal symptoms in patients after non‐oncological colorectal resections. For a further detail on the role of stoma formation, see ‘Part I—Sequelae to oncological diseases’, of these guidelines.

**Recommendations for the second‐line treatment of gastrointestinal symptoms after non‐oncological colorectal resections | Surgical interventions**

**Table:** 

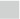	Surgical interventions can be considered for an individual patient if conservative first and/or second‐line treatments have failed to reach sufficient improvement of gastro‐intestinal symptoms after non‐oncological colorectal resections.
	*Good Practice Statement, ungraded*
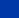	Sacral neuromodulation can be considered as surgical treatment for faecal incontinence.
	*Very low level of evidence*
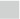	A stoma can be considered in patients with faecal incontinence after non‐oncological (colo)rectal resections for those patients who have refractory symptoms.
	*Good Practice Statement, ungraded*
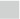	Pre‐operative stoma education can be considered in patients who will undergo stoma formation.
	*Good Practice Statement, ungraded*

## DISCUSSION

This is an up‐to‐date, European, multidisciplinary clinical practice guideline for the assessment and management of gastrointestinal symptoms after non‐oncological colorectal resections. We incorporated 20 recommendations addressing the assessment and management of gastrointestinal symptoms after non‐oncological colorectal resections. Subsequently, a treatment algorithm (Figure 1) has been developed to offer a visual representation of the key recommendations. The development of this algorithm involved visually representing the formulated recommendations, which were derived from a systematic and rigorous review of the best available evidence. In instances where literature was lacking, recommendations (or Good Practice Statements) were informed by the expert opinions of the GDG members involved. The guideline development group recommends using this algorithm as a guide alongside the main text to assist readers navigate through potential diagnostic modalities or treatment options. To the best of our knowledge, this is the first multidisciplinary guideline specifically addressing gastrointestinal symptoms after non‐oncological colorectal resections.

We want to highlight the potential benefits for patients of engaging a diverse group of specialised healthcare professionals to provide support, for both patients and their families in the challenging management of post‐surgical gastrointestinal symptoms. This collaborative effort may include contributions from gastroenterologists, colorectal surgeons, general practitioners, specialised nurses including ostomy care nurses, urologists, gynaecologists, pelvic floor physiotherapists, social workers, psychologists, dietitians, and even participation from patient support groups and associations.^41^, ^42^ The diverse expertise and insights provided by this multidisciplinary team could lead to an improvement of QoL for these patients.^43^

The main strength of this guideline lies in the multidisciplinary and international approach, leveraging diverse perspectives on various treatment options. The active involvement of patients in developing this guideline ensures that all crucial aspects and key perspectives are considered. The scope of this guideline did not include preventive measures, including considering options prior to treatment. We conducted a systematic literature review and included the best available evidence. To improve the quality and thus the strength of the recommendations, future prospective trials of high quality are needed. Therefore, we would like to highlight that the evidence regarding this specific topic is very scarce.

All UEG channels will be utilised for the widespread dissemination of this guideline, including the UEG Guideline app. The guideline and treatment algorithm will be available in the, including the UEG Guideline app, with very minimal resources required to access these documents. Additional support from all participating societies will contribute to the broad distribution and implementation of the guideline. Local adaptation of this guideline, in collaboration with local stakeholders, could potentially help overcome economic or infrastructural challenges in the implementation. This guideline will be updated in consultation with the UEG Quality of Care committee, subject to the allocation of sufficient funding. Any updates will follow a systematic and methodologically rigorous approach carried out in collaboration with the UEG and other participating associations. The literature search will be repeated annually in order to identify new evidence. In case this new evidence would substantially impact the recommendations in this guideline, then an update will be provided.

## AUTHOR CONTRIBUTIONS

Stephanie O. Breukink, Deena Harji and Daniel Keszthelyi were the lead authors responsible for the assembly of the GDG and drafting of the guidelines protocol. The initial list of research questions and core outcomes to be covered by these guidelines were drafted by Stephanie O. Breukink, Deena Harji, Daniel Keszthelyi, Anke H. C. Gielen and methodologist Jos Kleijnen. All research questions and intended outcomes were revised by all GDG members. The literature search was conducted by Anke H. C. Gielen under supervision of the methodologist Jos Kleijnen. Screening and selection of the articles was independently performed by Anke H. C. Gielen and Coco Smit. Data extraction was performed by Anke H. C. Gielen and verified by Coco Smit. The quality of evidence of the included articles was systematically appraised according to the GRADE method by Anke H. C. Gielen and verified by Jos Kleijnen. All GDG members and the external reviewer (Marc Gladman) discussed the results and reached a consensus on the recommendations. Lead authors Stephanie O. Breukink, Daniel Keszthelyi and Anke H. C. Gielen drafted this manuscript, which was reviewed, revised, and approved by GDG members mentioned above.

## CONFLICT OF INTEREST STATEMENT

The authors would like to report the following potential conflict(s) of interest: D. Keszthelyi, ZonMw (Dutch government), Dutch Foundation for Gastroenterology (MLDS), Allergan, Rome Foundation Horizon 2020, speaking at event Falk Foundation; J. Melenhorst, ZonMw (Dutch government); S. O. Breukink, ZonMw (Dutch government), Nationale Fonds tegen Kanker (National fund against Cancer) C. Kontovounisios, stakeholder One Welbeck hospital; A. Weimann, receipt of research supports B. Braun, Mucos and Seca, speaker at events of Abbott, Baxter, B. Braun, Fresenius Kabi and the Falk Foundation; H. Mohan, International Medical Robotics Academy consultation fees; J. Kleijnen, ESCP consultation fees, owner of Kleijnen Systematic Reviews Ltd; M. Gladman, grant from the Colorectal Surgical Society of Australia & New Zealand.

## DISCLAIMER

These guidelines have been developed with reasonable care and with the best of knowledge available to the authors at the time of preparation. They are intended to assist healthcare professionals and allied healthcare professionals as an educational tool to provide information that may support them in providing care to patients. Patients or other community members using these guidelines should do so only after consulting a health professional and should not construe these guidelines as professional medical advice. These guidelines must not substitute seeking professional medical and health advice from a health professional.

These guidelines may not apply to all situations and should be interpreted in the light of specific clinical situations and resource availability. It is up to every clinician to adapt these guidelines to local regulations and to each patient's individual circumstances and needs. The information in these guidelines should not be relied upon as being complete, current or accurate, nor should it be regarded as including all appropriate treatments or methods of care or as a legal standard of care. The development of this guideline was made possible by a grant from UEG (‘UEG Activity Grant 2022’) that was supplemented by the ESCP.

UEG makes no warranty, express or implied, with respect to these guidelines and shall not be held liable for any damages resulting from the application of these guidelines, in particular for any loss or damage (direct or indirect) resulting from treatment based on the guidance provided herein.

UEG cannot not be held liable, to the extent permitted by applicable law, for any content available on external websites, which can be reached by using the links provided herein.

## Supporting information

Supporting Information S1

Supporting Information S2

## Data Availability

Data sharing is not applicable to this guideline, since no new data were created or analysed in this project. All results as presented in this manuscript were directly derived from data as presented in the original articles. These are all included in the list of references.

## Members of the guideline development group

Stavros A. Antoniou: Department of Surgery, Papageorgiou General Hospital, Thessaloniki, Greece.

Geerard L. Beets: School for Oncology and Reproduction (GROW), Maastricht University, Maastricht, The Netherlands; Department of Surgery, The Netherlands Cancer Institute, Amsterdam, The Netherlands.

Stephanie O. Breukink: Department of Surgery, Maastricht University (Maastricht University, including Maastricht UMC+), Maastricht, The Netherlands; School of Nutrition and Translational Research in Metabolism (NUTRIM), Maastricht University, Maastricht, The Netherlands; School for Oncology and Reproduction (GROW), Maastricht University, Maastricht, The Netherlands.

Suzanne Dore: Patient Advisory Board Representative, UK.

Asbjørn M. Drewes: Department of Gastroenterology & Hepatology, Mech‐Sense, Aalborg University Hospital, Aalborg, Denmark; Danish Cancer Society Centre for Research on Survivorship and Late Adverse Effects After Cancer in the Pelvic Organs.

Hannah Garside: Department of Colorectal Surgery, Western General Hospital, Edinburgh, UK.

Marc A. Gladman: Faculty of Health & Medical Sciences, Adelaide Medical School, The University of Adelaide, Adelaide, Australia.

Deena Harji: Department of Colorectal Surgery, Manchester University NHS Foundation Trust, Manchester, UK.

Goran Hauser: Faculty of Medicine University of Rijeka, Department of Gastroenterology, Clinical Hospital Centre Rijeka, Rijeka, Croatia.

Therese Juul: Danish Cancer Society Centre for Research on Survivorship and Late Adverse Effects After Cancer in the Pelvic Organs; Department of Surgery, Aarhus University Hospital, Aarhus, Denmark.

Daniel Keszthelyi: School of Nutrition and Translational Research in Metabolism (NUTRIM), Maastricht University, Maastricht, The Netherlands; Department of Internal Medicine, Division of Gastroenterology‐Hepatology, Maastricht University Medical Centre, Maastricht, The Netherlands.

Jos Kleijnen: School for Oncology and Reproduction (GROW), Maastricht University, Maastricht, The Netherlands.

Christos Kontovounisios: Department of Surgery, Papageorgiou General Hospital, Thessaloniki, Greece; Department of Colorectal Surgery, Chelsea and Westminster Hospital NHS Foundation Trust, London, UK.

Laura Lorenzon: General Surgery Unit, Fondazione Policlinico Universitario ‘A. Gemelli’—IRCCS, Rome, Italy.

Lisa Massey: Department of Colorectal Surgery, Nottingham University Hospitals, Nottingham, UK.

Jarno Melenhorst: Department of Surgery, Maastricht University (Maastricht University, including Maastricht UMC+), Maastricht, The Netherlands; School of Nutrition and Translational Research in Metabolism (NUTRIM), Maastricht University, Maastricht, The Netherlands; School for Oncology and Reproduction (GROW), Maastricht University, Maastricht, The Netherlands.

Helen M. Mohan: Department of Colorectal Surgery, Western General Hospital, Edinburgh, UK.

Jean Muris: Department of General Practice, Care and Public Health Research Institute, Maastricht University, Maastricht, The Netherlands.

Coco Smit: Faculty of Health, Maastricht University, Medicine and Life Sciences, Maastricht, The Netherlands.

Yvonne Tillotson: Patient Advisory Board Representative, The Netherlands.

Arved Weimann: Department of General, Visceral and Oncological Surgery, St. George Hospital, Leipzig, Germany.

Marco Zelic: Department of Abdominal Surgery, Clinical Hospital Centre Rijeka, Rijeka, Croatia.
