# Supplementary material for: Guideline for the assessment and management of gastrointestinal symptoms following colorectal surgery—A UEG/ESCP/EAES/ESPCG/ESPEN/ESNM/ESSO collaboration. Part II—Good practice guidance | sequelae to benign diseases
Source: United European Gastroenterol J. 2024 Sep 14;12(8):1004–15. doi: 10.1002/ueg2.12659 (PMC11485301; doi:10.1002/ueg2.12659)
Supplement: Supplementary file 1 — Supporting Information S1 [file UEG2-12-1004-s001.docx]

**Appendix 1 – Methods**

These guidelines have been developed following the UEG Guideline for Guidelines, adhering to the AGREE II reporting framework, and adopting a systematic, methodologically rigorous and evidence-based approach [1, 2].

**Setting the scope**

The goal of this project was to create an up-to-date joint European, multidisciplinary guideline on the assessment and management of gastrointestinal symptoms after both oncological and benign colorectal surgery. These guidelines have been created in a collaboration with patients and members of the United European Gastroenterology (UEG), European Society of Coloproctology (ESCP), European Association of Endoscopic Surgery (EAES), European Society for Primary Care Gastroenterology (ESPCG), European Society for Clinical Nutrition and Metabolism (ESPEN), European Society of Neuro-gastroenterology and Motility (ESNM) and the European Society of Surgical Oncology (ESSO).

These guidelines provide guidance on the added value of diagnostic modalities and the effectiveness of treatment options for gastrointestinal symptoms following non-oncological colorectal resections. The guideline contains the following chapters:

- Diagnosis
- First line treatment
- Second line treatment: Non-surgical interventions
- Second line treatment: Surgical interventions

These guidelines are intended for use by all healthcare professionals treating patients experiencing gastrointestinal symptoms following non-oncological colorectal resections (e.g. nurses, general practitioners, gastroenterologists, colorectal surgeons, etc.). They also serve as a source of information for patients seeking knowledge about the diagnosis and treatment options for their gastrointestinal symptoms.

**Creation of the guideline development group (GDG)**

The steering committee, initiating this guideline project, consisted of four members (SB, DK, AG and DH). These four individuals consulted methodologists, representatives of different stakeholders and other potential users. Additionally, they informed and recruited the (potential) members of the Guideline Development Group (GDG). These guidelines were created by a multidisciplinary GDG, which consisted of 23 members from 7 European countries (i.e. Croatia, Denmark, Germany, Greece, Italy, The United Kingdom and The Netherlands). Therefore, we will adhere to the term ‘GDG’ in the main body text, instead of referring to the steering committee.

Several factors were considered in composing the GDG. First of all, all clinical members had to have an affinity with the diagnosis and treatment of gastrointestinal symptoms after colorectal surgery, considering that clinical expertise on the subject is of vital importance. Furthermore, the GDG had to be multidisciplinary. The GDG consisted of 11 colorectal surgeons (CK, AW, HM, LM, SA, GB, LL, JM, MZ, SB and DH), recruited from the ESCP, ESNM, ESPEN, ESSO and EAES, 3 gastroenterologists (AMD, GH, and DK) recruited from the ESCP and ESNM, one general practitioner (JM) recruited from the ESPCG, one methodologist with extensive experience in guideline development (JK), one INGUIDE (International Guideline Development Credentialing & Certification Programme) certified expert (LM), two experience experts as patient member (YT and SD), one independent researcher (CS) and one PhD candidate (AG). Distribution of geographical location between GDG members was aimed for, however expertise was decisive.

In addition to the specialists who were a part of the GDG, an Australian colorectal surgeon was on board as an external advisor (MG) in order to improve the quality of this guideline. The external reviewer, who had not been previously involved in the work process, conducted an independent assessment of the methodological quality of the process and reviewed the content of the guidelines. The GDG considered results of this external review in forming final recommendations. Meetings with the complete GDG took place roughly every 3-4 months over the course of a year to discuss the advancement of the guidelines.

**Table 1 –** Guideline Development Group (GDG)

| **Guideline Member** | **Function** | **Country** |
| --- | --- | --- |
| Stavros Antoniou | Colorectal surgeon | Greece |
| Geerard Beets | Colorectal surgeon | The Netherlands |
| Stephanie Breukink | Colorectal surgeon | The Netherlands |
| Deena Harji | Colorectal surgeon | The United Kingdom |
| Christos Kontovounisios | Colorectal surgeon | Greece |
| Laura Lorenzon | Colorectal surgeon | Italy |
| Lisa Massey | Colorectal surgeon | The United Kingdom |
| Jarno Melenhorst | Colorectal surgeon | The Netherlands |
| Helen Mohan | Colorectal surgeon | The United Kingdom |
| Arved Weimann | Colorectal surgeon | Germany |
| Marko Zelic | Colorectal surgeon | Croatia |
| Hannah Garside | Junior Doctor \| Surgical department | The United Kingdom |
| Goran Hauser | Gastroenterologist | Croatia |
| Daniel Keszthelyi | Gastroenterologist | The Netherlands |
| Asbjørn Mohr Drewes | Gastroenterologist | Denmark |
| Jean Muris | General Practitioner | The Netherlands |
| Therese Juul | Specialised nurse | Denmark |
| Jos Kleijnen | Methodologist | The United Kingdom |
| Yvonne Tillotson | Patient Member | The Netherlands |
| Suzanne Dore | Patient Member | The United Kingdom |
| Coco Smit | Independent researcher | The Netherlands |
| Marc Gladman | Gastroenterologist \| external reviewer | Australia |
| Anke Gielen | PhD Candidate | The Netherlands |

**Formulating review questions (PICO)**

The GDG developed review questions to evaluate the key issues specified in the scope. To assess the effectiveness of interventions, the GDG formulated review questions using the PICO (populations, intervention, comparator and outcome) framework. For other types of questions (e.g. regarding diagnosis), similar frameworks were applied.

The GDG identified the chapters to be included in the guidelines and decided on the intended outcomes through group discussion. The review questions were then clustered into different categories (i.e. diagnosis, first line treatment and both surgical and non-surgical interventions for second-line treatment).

To streamline the process, subgroups were formed within the GDG, with each subgroup assigned to focus on a specific chapter. This approach allowed for a more efficient and effective development of the guidelines.

**Table 2 –** formulating a review question on the effectiveness of an intervention using the PICO framework.

| Population | What is the best way to describe the population we are interested in? |
| --- | --- |
| Intervention | Which interventions or treatments should or could be used?  (Or which diagnostic modalities need to be considered) |
| Comparator | Are there any alternatives available to the intervention being considered? |
| Outcome | Which outcomes should be considered in order to assess the effect of the proposed intervention? Which outcomes are most important to both patients and healthcare professionals? |

A set of provisional outcomes was created prior to the first GDG meeting by AG, based on a scoping literature search to encourage discussion regarding relevant outcomes. During this initial meeting, the GDG decided on a final set of intended outcomes. A list of the five most important outcomes is demonstrated in Table 3, ranked in order of importance. This list of outcomes was used to determine which results should eventually be reported in these guidelines, and the review questions were formulated based on these outcomes. By aligning the review questions with the intended outcomes, the GDG aimed to develop guidelines that would have a meaningful impact on patient outcomes and clinical practice.

The outcomes which were included were assessed for quality of evidence using the GRADE (Grading of Recommendations, Assessment, Development and Evaluations) assessment criteria [3, 4]. The quality of evidence in combination with the results of the study determine the strength of the evidence, which in turn determines the wording used in the formulated recommendations.

**Table 3 –** Set of critical and important outcomes, ranked order of importance

| **Outcome measures** |
| --- |
| Overall Quality of Life (QoL) |
| Involuntary loss of stool (i.e. soiling or faecal incontinence).  We will address this topic, however we will refer to the up-to-date UEG guideline on faecal incontinence for further elaboration [5]. |
| Abdominal pain |
| Alteration in bowel habit (i.e. constipation, frequency of diarrhoea, incomplete evacuation; fragmentation or clustering of stool) |
| Urgency |
| *The cost-effectiveness of suggested treatments or measures will be taken into account* |

**Literature search**

The review questions provided a framework for conducting the literature searches. The searches were carried out in MEDLINE (Ovid), EMBASE (Ovid), Ksrevidence and the Cochrane Database of Systematic Reviews. The search terms included colorectal surgery and gastrointestinal symptoms as well as any synonyms and variations of these terms. The search covered all relevant studies published between January 1^st^ 2000 and May 1^st^ 2023. The full search strategy is presented at the bottom of this document. The searches in the databases were supplemented by checking references in the reviews and primary studies found. Any additional relevant studies that the GDG was aware of were also included in the review.

We included the studies with the best available evidence for each predefined research question. Whenever evidence of a higher level was accessible, we excluded lower levels of evidence, unless they contributed insights into new (sub)topics. If systematic reviews and/or meta-analyses were available, we utilized these, along with any previously omitted RCT’s to address the research question. In the absence of systematic reviews and/or meta-analyses, RCT’s were included. If no reviews or RCT’s were available, observational studies with a control group were included. When none of the aforementioned study designs were available, we relied on expert opinions.

The search resulted in 25.863 records prior to de-duplication and 21.517 after de-duplication (13.513 oncological, 8004 benign). Two independent reviewers (AG and CS) screened the potentially eligible studies for inclusion. Any disagreements were discussed and resolved between the two reviewers. After title and abstract review 88 (67 oncological, 21 benign) records were subjected to full article review of which 35 articles were included in these guidelines. 26 articles were included in the oncological guideline, and 9 articles in the guideline on gastrointestinal symptoms after colorectal resections for benign indications.

**Reviewing research evidence**

Data was extracted by one reviewer (AG) and subsequently checked by a second reviewer (CS). The data extracted for each included study was aligned with the predefined set of outcomes (Table 3). Our primary objective involved assessing differences in treatment effects between baseline and post-treatment within the treatment group, compared to the control group if the data allowed us to do so.

Considering substantial variations in healthcare implementation costs and differences in coverage by health insurance across Europe, we have incorporated cost considerations only to a limited extent in our evidence review to inform the guideline.

**Wording recommendations using GRADE**

Following a complete review of the evidence, a meticulous systematic quality-assessment was conducted for each research question using the GRADE approach [1,2]. This method ensured that the quality of evidence was evaluated across all included studies. The grading of evidence quality was categorized into levels as ‘high’, ‘moderate’, ‘low’, ‘very low’ as elaborated in Table 4. In instances were even ‘very low’ levels of evidence were unavailable, expert opinion was applied and registered as level of evidence.

The phrasing of each recommendation was dependent upon the corresponding level of quality of evidence, as outlined in Table 5. Recommendations associated with the ‘expert opinion’ level of evidence were drafted with flexibility and left up to the discretion of the GDG and experts. These recommendations are referred to as ‘Good Practice Statements’. Following the formulation of recommendations based on the evidence review GRADE-based assessment of evidence quality, these recommendations were discussed within the GDG. The GDG retained the authority to upgrade or downgrade recommendations if deemed necessary based on their clinical expertise. Such adjustments, if made, were explicitly indicated alongside the respective recommendation. The colour scheme was adjusted accordingly, aligning each colour with its respective level of evidence after any upgrades or downgrades. All members of the GDG were invited to two online consensus meetings, where the wording of every recommendation and the content of the evidence to decision frameworks was discussed in detail. Consensus within the GDG was reached unanimously for all recommendations after the second consensus meeting. Therefore, this informal consensus methodology was deemed sufficient, in accordance with the UEG Guideline for Guidelines [1]. The GRADE evidence-to-decision framework was used to document the discussions within the GDG in a comprehensive manner [6]. Following the advice of our methodologist, we have created several evidence-to-decision frameworks for both parts of this guideline. The most relevant and debatable recommendations were selected by the steering committee and used for these frameworks. The overall ranking ‘strong’ or ‘conditional’ recommendation is also presented at the bottom of this framework. The evidence-to-decision frameworks were composed by one researcher (AG) and verified by both the methodologist (JK) and all other members of the GDG.

**Table 4** – GRADE classification of quality of evidence [3]

| High | Further research is very unlikely to change our confidence in the estimate of effect. |
| --- | --- |
| Moderate | Further research is likely to have an important impact on our confidence in the estimate of effect and may change the estimate. |
| Low | Further research is very likely to have an important impact on our confidence in the estimate of effect and is likely to change the estimate. |
| Very low | Any estimate of effect is very uncertain. |

**Table 5** – Wording of recommendations according to GRADE classification [3, 4]

|  | Quality of evidence according to GRADE | Wording recommendation |
| --- | --- | --- |
|  | Moderate level | ‘’Should be used’’ |
|  | Low level | ‘’Could be used’’ |
|  | Very low level  Good Practice Statement | ‘’Can be considered’’ |

**References**

1. Boltin D, Lambregts DM, Jones F, Siterman M, Bonovas S, Cornberg M, et al. UEG framework for the development of high-quality clinical guidelines. United European Gastroenterol J. 2020;8(8):851-64.

2. Brouwers MC, Kho ME, Browman GP, Burgers JS, Cluzeau F, Feder G, et al. AGREE II: advancing guideline development, reporting and evaluation in health care. Cmaj. 2010;182(18):E839-E42.

3. Group GW. Grading quality of evidence and strength of recommendations. Bmj. 2004;328(7454):1490.

4. Santesso N, Glenton C, Dahm P, Garner P, Akl EA, Alper B, et al. GRADE guidelines 26: informative statements to communicate the findings of systematic reviews of interventions. Journal of clinical epidemiology. 2020;119:126-35.

5. Assmann SL, Keszthelyi D, Kleijnen J, Anastasiou F, Bradshaw E, Brannigan AE, et al. Guideline for the diagnosis and treatment of Faecal Incontinence—A UEG/ESCP/ESNM/ESPCG collaboration. United European Gastroenterol J. 2022;10(3):251-86.

6. Alonso-Coello P, Schünemann HJ, Moberg J, Brignardello-Petersen R, Akl EA, Davoli M, et al. GRADE Evidence to Decision (EtD) frameworks: a systematic and transparent approach to making well informed healthcare choices. 1: Introduction. bmj. 2016;353.
