# Supplementary material for: Guideline for the assessment and management of gastrointestinal symptoms following colorectal surgery—A UEG/ESCP/EAES/ESPCG/ESPEN/ESNM/ESSO collaboration. Part II—Good practice guidance | sequelae to benign diseases
Source: United European Gastroenterol J. 2024 Sep 14;12(8):1004–15. doi: 10.1002/ueg2.12659 (PMC11485301; doi:10.1002/ueg2.12659)
Supplement: Supplementary file 2 — Supporting Information S2 [file UEG2-12-1004-s002.docx]

| ***Problem***: Gastrointestinal symptoms  ***Option***: Assessment of post-operative symptoms (including pain)  ***Comparison***: No assessment of post-operative symptoms (including pain)  ***Setting***: In hospital or primary care | | | ***Background and Objective***: The evaluation of every problem, should start with a detailed patient history. Understanding post-operative symptoms in patients undergoing colorectal surgery is a crucial starting point for optimising patient care and outcomes. This question addresses whether the assessment of post-operative symptoms should be performed in patients experiencing gastrointestinal symptoms after non-oncological colorectal resections. | |
| --- | --- | --- | --- | --- |
|  | **Criteria** | **Judgements** | **Research evidence** | **Additional considerations** |
| Problem | Is there a problem priority? | ○ No  ○ Probably no  ○ Uncertain  ○ Probably yes  ● Yes  ○ Varies | Benign colorectal resections are performed to address a spectrum of non-oncological conditions, including diverticulitis, inflammatory bowel disease (IBD), polyposis, functional bowel disorders or endometriosis. While the surgical procedure mirrors that of oncological cases, the primary distinction lies in the preservation of lymph nodes in these non-oncological resections [1]. Nevertheless, irrespective of the underlying condition, any type of colorectal resection could have an adverse impact on the patient’s bowel function [2-6]. Bowel dysfunction can manifest in a wide spectrum of symptoms, including urgency complaints, constipation, faecal incontinence and/or abdominal pain, all of which require very different management strategies [8, 9]. Understanding the range of gastrointestinal outcomes, their impact and consequences following non-oncological colorectal resections can improve quality of life in this cohort of patients. Therefore, the GDG, in collaboration with patient representatives, has identified the issue of gastrointestinal symptoms after colorectal surgery as a priority. |  |
| Benefits & harms of the options | What is the overall certainty of this evidence? | ○ No included studies  ○ Very low  ● Low  ○ Moderate  ○ High | \| **No. of studies** \| **Design** \| **Limitations (RoB)** \| **Indirectness of patients, intervention and comparator** \| **Inconsistency** \| **Imprecision** \| **Other considerations** \| **Certainty of evidence** \| \| --- \| --- \| --- \| --- \| --- \| --- \| --- \| --- \| \| 1 \| Cohort study \| Low \| Only available for pouch patients \| None \| None \| None \| 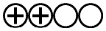 \| |  |
|  | Is there important uncertainty about how much people value the main outcomes? | ○ Yes  ○ Possibly  ● Probably not  ○ No  ○ Unknown | GDG members, including patient representatives, noted that there is no important variability or uncertainty in patients’ values and preferences. Specifically, patients would highly prioritise a reduction in the burden of disease associated with gastrointestinal symptoms after non-oncological colorectal resections. |  |
|  | Are the desirable anticipated effects large? | ○ No  ○ Probably no  ○ Uncertain  ● Probably yes  ○ Yes  ○ Varies | By adequately assessing the experienced symptoms, healthcare professionals can eventually identify potential complications, tailor interventions, and enhance patient satisfaction and quality of life. Healthcare professionals have to understand what it is that is troubling the patient before starting any interventions and evaluating the effect of the treatments. |  |
|  | Are the undesirable anticipated effects small? | ○ No  ○ Probably no  ○ Uncertain  ● Probably yes  ○ Yes  ○ Varies | There are no data available on the potential harms of the assessment of post-operative symptoms after colorectal surgery. | The GDG reported only potential health benefits of an adequate assessment of experienced symptoms. |
|  | Are the desirable effects large relative to undesirable effects? | ○ No  ○ Probably no  ○ Uncertain  ● Probably yes  ○ Yes  ○ Varies | As stated before, there are no data available on the potential harms of the assessment of post-operative symptoms after colorectal surgery. Therefore, the GDG reckons that the desirable effects are large relative to the potential undesirable effects. |  |
| Resource use | Are the required resources small? | ○ No  ○ Probably no  ○ Uncertain  ○ Probably yes  ● Yes  ○ Varies | The only resources required for the assessment of post-operative symptoms include the time of a dedicated healthcare professional and a private space for this conversation, as is already standard daily clinical practice in every consultation in the outpatient clinic. |  |
|  | Is the incremental cost small relative to the net benefits? | ○ No  ○ Probably no  ○ Uncertain  ● Probably yes  ○ Yes  ○ Varies | No evidence identified specific to the assessment of gastrointestinal symptoms after oncological colorectal resections. The GDG considered the incremental relatively small to the potential benefits of improved understanding and patient-healthcare professional relationships. | No formal cost-effectiveness analysis has been conducted since the limited data identified. |
| Equity | What would be the impact on health equity? | ○ Increased  ● Probably increased  ○ Uncertain  ○ Probably reduced  ○ Reduced  ○ Varies | No evidence identified specific to the assessment of post-operative symptoms on health in patients after non-oncological colorectal resections. Considering the fact that no potential harmful effects are identified, the GDG reckons that the general health equity would probably be increased. |  |
| Acceptability | Is the intervention acceptable to patients, their caregivers and healthcare providers? | ○ No  ○ Probably no  ○ Uncertain  ● Probably yes  ○ Yes  ○ Varies | The GDG did not consider this as an intervention, as this question describes a detailed patient history, which already is considered as standard daily clinical practice. |  |
| Feasibility | Is the intervention feasible to implement? | ○ No  ○ Probably no  ○ Uncertain  ● Probably yes  ○ Yes  ○ Varies | No evidence identified specific to the feasibility of the assessment of post-operative symptoms after oncological colorectal resections. The GDG considered the intervention as a feasible option. |  |
| Overall ranking | | Strong recommendation | | |

**1. The assessment of post-operative symptoms**

| ***Problem***: Gastrointestinal symptoms  ***Option***: Colonoscopy  ***Comparison***: No colonoscopy  ***Setting***: In hospital or primary care | | | ***Background and Objective***: After a detailed patient history, there are a variety of diagnostic modalities which can be used to assess and evaluate gastrointestinal symptoms, including colonoscopy, anorectal manometry or endoanal ultrasound. This question addresses whether a colonoscopy should be considered as additional diagnostic modality in patients experiencing gastrointestinal symptoms after non- oncological colorectal resections. | |
| --- | --- | --- | --- | --- |
|  | **Criteria** | **Judgements** | **Research evidence** | **Additional considerations** |
| Problem | Is there a problem priority? | ○ No  ○ Probably no  ○ Uncertain  ○ Probably yes  ● Yes  ○ Varies | Benign colorectal resections are performed to address a spectrum of non-oncological conditions, including diverticulitis, inflammatory bowel disease (IBD), polyposis, functional bowel disorders or endometriosis. While the surgical procedure mirrors that of oncological cases, the primary distinction lies in the preservation of lymph nodes in these non-oncological resections [1]. Nevertheless, irrespective of the underlying condition, any type of colorectal resection could have an adverse impact on the patient’s bowel function [2-6]. Bowel dysfunction can manifest in a wide spectrum of symptoms, including urgency complaints, constipation, faecal incontinence and/or abdominal pain, all of which require very different management strategies [8, 9]. Understanding the range of gastrointestinal outcomes, their impact and consequences following non-oncological colorectal resections can improve quality of life in this cohort of patients. Therefore, the GDG, in collaboration with patient representatives, has identified the issue of gastrointestinal symptoms after colorectal surgery as a priority. |  |
| Benefits & harms of the options | What is the overall certainty of this evidence? | ○ No included studies  ● Very low  ○ Low  ○ Moderate  ○ High | \| **No. of studies** \| **Design** \| **Limitations (RoB)** \| **Indirectness of patients, intervention and comparator** \| **Inconsistency** \| **Imprecision** \| **Other considerations** \| **Certainty of evidence** \| \| --- \| --- \| --- \| --- \| --- \| --- \| --- \| --- \| \| 2 \| Systematic Review \| 1x unclear, 1x high \| Only available for pouch patients \| None \| None \| None \| **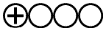** \| |  |
|  | Is there important uncertainty about how much people value the main outcomes? | ○ Yes  ○ Possibly  ● Probably not  ○ No  ○ Unknown | GDG members, including patient representatives, noted that there is no important variability or uncertainty in patients’ values and preferences. Specifically, patients would highly prioritise a reduction in the burden of disease associated with gastrointestinal symptoms after non-oncological colorectal resections. |  |
|  | Are the desirable anticipated effects large? | ○ No  ○ Probably no  ○ Uncertain  ● Probably yes  ○ Yes  ○ Varies | It is essential to rule out anatomical causes like anastomotic stenosis or pouch-related complications as underlying factors for the gastrointestinal symptoms. Early detection of these mechanical issues holds large anticipated effects, allowing for timely intervention and accurate treatment of the underlying pathology. |  |
|  | Are the undesirable anticipated effects small? | ○ No  ● Probably no  ○ Uncertain  ○ Probably yes  ○ Yes  ○ Varies | Potential harmful and undesirable effects of a colonoscopy have been reported. Possible complications include cardiopulmonary complications of the sedation, colonic perforation, haemorrhage, infection, (temporary) abdominal pain or discomfort, even mortality has been reported as a rare but possible complication [10]. |  |
|  | Are the desirable effects large relative to undesirable effects? | ○ No  ○ Probably no  ○ Uncertain  ● Probably yes  ○ Yes  ○ Varies | Even though complications are rare, they can be severe. Nonetheless, the anticipated benefits of early detection of the underlying cause of the symptoms outweigh the potential risks of complications for a colonoscopy. Therefore, the GDG reckons that the desirable effects are large relative to the potential undesirable effects. |  |
| Resource use | Are the required resources small? | ○ No  ● Probably no  ○ Uncertain  ○ Probably yes  ○ Yes  ○ Varies | The resources required for a colonoscopy vary depending on whether it is performed under sedation or general anaesthesia. Additionally, each case necessitates functioning equipment, a skilled professional to conduct the colonoscopy, specialized assisting nurses, and, of course, bowel preparation by the patients themselves in the days leading up to the procedure. |  |
|  | Is the incremental cost small relative to the net benefits? | ○ No  ○ Probably no  ● Uncertain  ○ Probably yes  ○ Yes  ○ Varies |  | No formal cost-effectiveness analysis has been conducted since the limited data identified. |
| Equity | What would be the impact on health equity? | ○ Increased  ● Probably increased  ○ Uncertain  ○ Probably reduced  ○ Reduced  ○ Varies | No evidence identified specific to dietary adjustments on health equity in patients after non-oncological colorectal resections. Considering the fact that early detection the underlying pathophysiological mechanism of the experienced symptoms outweighs the potential harmful effects of complications after a colonoscopy, the GDG reckons that the general health equity would probably be increased. |  |
| Acceptability | Is the intervention acceptable to patients, their caregivers and healthcare providers? | ○ No  ○ Probably no  ○ Uncertain  ● Probably yes  ○ Yes  ○ Varies | No evidence identified specific to the acceptability of a colonoscopy as additional diagnostic modality in patients with gastrointestinal symptoms after oncological colorectal resections. However, it is a common procedure used for screening, diagnosis and monitoring of colorectal conditions in daily clinical practice. The GDG considered the intervention as a possibly acceptable option to the key stakeholders. | The acceptability of a colonoscopy as a diagnostic modality always depends on the specific clinical situation and the individual patient. |
| Feasibility | Is the intervention feasible to implement? | ○ No  ○ Probably no  ○ Uncertain  ● Probably yes  ○ Yes  ○ Varies | No evidence identified specific to the feasibility of a colonoscopy as additional diagnostic modality for gastrointestinal symptoms after non-oncological colorectal resections. The GDG considered the intervention as a feasible diagnostic modality if sufficient funding and resources are available at a local treatment facility. |  |
| Overall ranking | | Conditional recommendation | | |

**2. Colonoscopy**

| ***Problem***: Gastrointestinal symptoms  ***Option***: Dietary adjustments  ***Comparison***: No dietary adjustments  ***Setting***: In hospital or primary care | | | ***Background and Objective***: In our society, an increasing number of individuals is obese and/.or maintains unhealthy lifestyle factors. Diet is a pivotal component within the multifactorial concept of lifestyle. It is well-established that diet and dietary changes significantly impact bowel habits, particularly in the context of inflammatory bowel syndrome (IBS) or inflammatory bowel disease (IBD) [1, 2]. This question addresses whether dietary adjustments should be performed in patients experiencing gastrointestinal symptoms after non-oncological colorectal resections. | |
| --- | --- | --- | --- | --- |
|  | **Criteria** | **Judgements** | **Research evidence** | **Additional considerations** |
| Problem | Is there a problem priority? | ○ No  ○ Probably no  ○ Uncertain  ○ Probably yes  ● Yes  ○ Varies | Benign colorectal resections are performed to address a spectrum of non-oncological conditions, including diverticulitis, inflammatory bowel disease (IBD), polyposis, functional bowel disorders or endometriosis. While the surgical procedure mirrors that of oncological cases, the primary distinction lies in the preservation of lymph nodes in these non-oncological resections [1]. Nevertheless, irrespective of the underlying condition, any type of colorectal resection could have an adverse impact on the patient’s bowel function [2-6]. Bowel dysfunction can manifest in a wide spectrum of symptoms, including urgency complaints, constipation, faecal incontinence and/or abdominal pain, all of which require very different management strategies [8, 9]. Understanding the range of gastrointestinal outcomes, their impact and consequences following non-oncological colorectal resections can improve quality of life in this cohort of patients. Therefore, the GDG, in collaboration with patient representatives, has identified the issue of gastrointestinal symptoms after colorectal surgery as a priority. |  |
| Benefits & harms of the options | What is the overall certainty of this evidence? | ○ No included studies  ○ Very low  ○ Low  ● Moderate  ○ High | \| **No. of studies** \| **Design** \| **Limitations (RoB)** \| **Indirectness of patients, intervention and comparator** \| **Inconsistency** \| **Imprecision** \| **Other considerations** \| **Certainty of evidence** \| \| --- \| --- \| --- \| --- \| --- \| --- \| --- \| --- \| \| 1 \| RCT \| Low \| Only available for pouch patients \| None \| None \| None \| **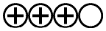** \| |  |
|  | Is there important uncertainty about how much people value the main outcomes? | ○ Yes  ○ Possibly  ● Probably not  ○ No  ○ Unknown | GDG members, including patient representatives, noted that there is no important variability or uncertainty in patients’ values and preferences. Specifically, patients would highly prioritise a reduction in the burden of disease associated with gastrointestinal symptoms after non-oncological colorectal resections. |  |
|  | Are the desirable anticipated effects large? | ○ No  ○ Probably no  ● Uncertain  ○ Probably yes  ○ Yes  ○ Varies | Anticipated positive outcomes may vary based on the specific dietary adjustments undertaken. Unfortunately, within this particular patient cohort, only a singular and highly specific dietary adjustment – probiotic administration – was identified. However, for patients with IBS/IBD who have not undergone resections, numerous potentially beneficial dietary interventions have been reported. This is why the GDG reckons that dietary adjustments can be considered as treatment for gastrointestinal symptoms after non-oncological colorectal resections. |  |
|  | Are the undesirable anticipated effects small? | ○ No  ○ Probably no  ○ Uncertain  ● Probably yes  ○ Yes  ○ Varies | No harmful effects or adverse events were reported for the administered probiotics. |  |
|  | Are the desirable effects large relative to undesirable effects? | ○ No  ○ Probably no  ○ Uncertain  ● Probably yes  ○ Yes  ○ Varies |  |  |
| Resource use | Are the required resources small? | ○ No  ○ Probably no  ○ Uncertain  ○ Probably yes  ● Yes  ○ Varies | No evidence identified specific to dietary adjustments as treatment for gastrointestinal symptoms after non-oncological colorectal resections. The GDG considered the incremental costs of dietary adjustments relatively small to the potential benefits. |  |
|  | Is the incremental cost small relative to the net benefits? | ○ No  ○ Probably no  ○ Uncertain  ● Probably yes  ○ Yes  ○ Varies |  | No formal cost-effectiveness analysis has been conducted since the limited data identified. |
| Equity | What would be the impact on health equity? | ○ Increased  ● Probably increased  ○ Uncertain  ○ Probably reduced  ○ Reduced  ○ Varies | No evidence identified specific to dietary adjustments on health equity in patients after non-oncological colorectal resections. Considering the fact that no potential harmful effects are identified, the GDG reckons that the general health equity would probably be increased. |  |
| Acceptability | Is the intervention acceptable to patients, their caregivers and healthcare providers? | ○ No  ○ Probably no  ○ Uncertain  ● Probably yes  ○ Yes  ○ Varies | No evidence identified specific to the acceptability of dietary adjustments on gastrointestinal symptoms after non-oncological colorectal resections. The GDG considered the intervention as a possibly acceptable option to the key stakeholders. |  |
| Feasibility | Is the intervention feasible to implement? | ○ No  ○ Probably no  ○ Uncertain  ● Probably yes  ○ Yes  ○ Varies | No evidence identified specific to the feasibility of dietary adjustments on gastrointestinal symptoms after non-oncological colorectal resections The GDG considered the intervention as a feasible treatment option if sufficient funding and resources are available at a local treatment facility in order to provide guidance to the patient, i.e. by a dietitian. |  |
| Overall ranking | | Strong recommendation | | |

**3. Dietary adjustments**

| ***Problem***: Gastrointestinal symptoms  ***Option***: Medication (i.e. stool bulking agents, laxatives, antidiarrheal medication and/or bile acid binders)  ***Comparison***: No medication  ***Setting***: In hospital | | | ***Background and Objective***: Many patients experience altered stool consistency and/or bowel habits after non-oncological colorectal resections. A medication-based treatment has the advantage that it is very minimally invasive, able to be adjusted rapidly and to vary within administered drugs (for instance laxatives after stool bulking agents). This question addresses whether medication (i.e. stool bulking agents such as psyllium, laxatives such as macrogol, antidiarrheal medication such as loperamide and/or bile acid binders such as cholestyramine) should be administered to patients experiencing gastrointestinal symptoms after oncological colorectal resections. | |
| --- | --- | --- | --- | --- |
|  | **Criteria** | **Judgements** | **Research evidence** | **Additional considerations** |
| Problem | Is there a problem priority? | ○ No  ○ Probably no  ○ Uncertain  ○ Probably yes  ● Yes  ○ Varies | Benign colorectal resections are performed to address a spectrum of non-oncological conditions, including diverticulitis, inflammatory bowel disease (IBD), polyposis, functional bowel disorders or endometriosis. While the surgical procedure mirrors that of oncological cases, the primary distinction lies in the preservation of lymph nodes in these non-oncological resections [1]. Nevertheless, irrespective of the underlying condition, any type of colorectal resection could have an adverse impact on the patient’s bowel function [2-6]. Bowel dysfunction can manifest in a wide spectrum of symptoms, including urgency complaints, constipation, faecal incontinence and/or abdominal pain, all of which require very different management strategies [8, 9]. Understanding the range of gastrointestinal outcomes, their impact and consequences following non-oncological colorectal resections can improve quality of life in this cohort of patients. Therefore, the GDG, in collaboration with patient representatives, has identified the issue of gastrointestinal symptoms after colorectal surgery as a priority. |  |
| Benefits & harms of the options | What is the overall certainty of this evidence? | ○ No included studies  ○ Very low  ● Low  ○ Moderate  ○ High | \| **No. of studies** \| **Design** \| **Limitations (RoB)** \| **Indirectness of patients, intervention and comparator** \| **Inconsistency** \| **Imprecision** \| **Other considerations** \| **Quality of evidence** \| \| --- \| --- \| --- \| --- \| --- \| --- \| --- \| --- \| \| 2 \| RCT \| Moderate \| None \| 1 paper on octreotide, 1 on calcium polycarbophil \| None \| None \| 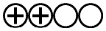 \| |  |
|  | Is there important uncertainty about how much people value the main outcomes? | ○ Yes  ○ Possibly  ● Probably not  ○ No  ○ Unknown | GDG members, including patient representatives, noted that there is no important variability or uncertainty in patients’ values and preferences. Specifically, patients would highly prioritise a reduction in the burden of disease associated with gastrointestinal symptoms after non-oncological colorectal resections. |  |
|  | Are the desirable anticipated effects large? | ○ No  ○ Probably no  ○ Uncertain  ○ Probably yes  ● Yes  ○ Varies | Considering the substantial impact that gastrointestinal symptoms can have on patients, such as limiting social activities due to the fear of experiencing episodes of faecal incontinence in public settings, the desirable anticipated effect of alleviating these symptoms is considered significant by the GDG. |  |
|  | Are the undesirable anticipated effects small? | ○ No  ○ Probably no  ○ Uncertain  ● Probably yes  ○ Yes  ○ Varies | Each type of medication carries potential side effects. Apart from allergic reactions, all conceivable side effects associated with these medications are temporary and reversible upon discontinuation. Switching to an alternative type of medication is always a viable option. |  |
|  | Are the desirable effects large relative to undesirable effects? | ○ No  ○ Probably no  ○ Uncertain  ● Probably yes  ○ Yes  ○ Varies |  |  |
| Resource use | Are the required resources small? | ○ No  ○ Probably no  ○ Uncertain  ○ Probably yes  ● Yes  ○ Varies | No evidence specific to the oncological patient population was identified. The GDG considered these generic types of medication as requiring minimal resources. |  |
|  | Is the incremental cost small relative to the net benefits? | ○ No  ○ Probably no  ○ Uncertain  ○ Probably yes  ● Yes  ○ Varies | The GDG reckoned that the incremental costs (medication vs. no medication) are small relative to the potential benefits. | Costs of medication may vary between countries or continents. |
| Equity | What would be the impact on health equity? | ○ Increased  ● Probably increased  ○ Uncertain  ○ Probably reduced  ○ Reduced  ○ Varies | No evidence identified specific to medication on health equity in CRC survivors. Considering the fact that only minor potential adverse events are identified, the GDG reckons that the general health equity would probably be increased. |  |
| Acceptability | Is the intervention acceptable to patients, their caregivers and healthcare providers? | ○ No  ○ Probably no  ○ Uncertain  ○ Probably yes  ● Yes  ○ Varies | No evidence identified specific to this patient population. The GDG considered the daily use of medication as an acceptable intervention. | Given the minimal costs and resources needed, payers are also thought to find this intervention acceptable. |
| Feasibility | Is the intervention feasible to implement? | ○ No  ○ Probably no  ○ Uncertain  ○ Probably yes  ● Yes  ○ Varies | No evidence identified specific to this patient population. The GDG considered the daily use of medication as a feasible intervention. |  |
| Overall ranking | | Conditional recommendation | | |

**4. Medication**

| ***Problem***: Gastrointestinal symptoms  ***Option***: Pelvic floor physiotherapy  ***Comparison***: No pelvic floor physiotherapy  ***Setting***: In hospital | | | ***Background and Objective***: Many patients experience altered stool consistency and/or bowel habits or even faecal incontinence after colorectal resections. Pelvic floor physiotherapy is a well-established course of treatment for patients experiencing faecal incontinence. Considering that many patients are also experiencing faecal incontinence after non-oncological colorectal resections, pelvic floor physiotherapy could be a treatment option worth considering for this particular patient population. This question addresses whether pelvic floor physiotherapy should be performed in patients experiencing faecal incontinence and/or other gastrointestinal symptoms after non-oncological colorectal resections. | |
| --- | --- | --- | --- | --- |
|  | **Criteria** | **Judgements** | **Research evidence** | **Additional considerations** |
| Problem | Is there a problem priority? | ○ No  ○ Probably no  ○ Uncertain  ○ Probably yes  ● Yes  ○ Varies | Benign colorectal resections are performed to address a spectrum of non-oncological conditions, including diverticulitis, inflammatory bowel disease (IBD), polyposis, functional bowel disorders or endometriosis. While the surgical procedure mirrors that of oncological cases, the primary distinction lies in the preservation of lymph nodes in these non-oncological resections [1]. Nevertheless, irrespective of the underlying condition, any type of colorectal resection could have an adverse impact on the patient’s bowel function [2-6]. Bowel dysfunction can manifest in a wide spectrum of symptoms, including urgency complaints, constipation, faecal incontinence and/or abdominal pain, all of which require very different management strategies [8, 9]. Understanding the range of gastrointestinal outcomes, their impact and consequences following non-oncological colorectal resections can improve quality of life in this cohort of patients. Therefore, the GDG, in collaboration with patient representatives, has identified the issue of gastrointestinal symptoms after colorectal surgery as a priority. | Very small cohort.  This evidence only applies to patients with a pouch. |
| Benefits & harms of the options | What is the overall certainty of this evidence? | ○ No included studies  ● Very low  ○ Low  ○ Moderate  ○ High | \| **No. of studies** \| **Design** \| **Limitations (RoB)** \| **Indirectness of patients, intervention and comparator** \| **Inconsistency** \| **Imprecision** \| **Other considerations** \| **Quality of evidence** \| \| --- \| --- \| --- \| --- \| --- \| --- \| --- \| --- \| \| 1 \| Cohort study \| Moderate \| Only available for pouch patients \| None \| None \| None \| **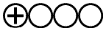** \| |  |
|  | Is there important uncertainty about how much people value the main outcomes? | ○ Yes  ○ Possibly  ● Probably not  ○ No  ○ Unknown | GDG members, including patient representatives, noted that there is no important variability or uncertainty in patients’ values and preferences. Specifically, patients would highly prioritise a reduction in the burden of disease associated with gastrointestinal symptoms after non-oncological colorectal resections. |  |
|  | Are the desirable anticipated effects large? | ○ No  ○ Probably no  ○ Uncertain  ○ Probably yes  ● Yes  ○ Varies | Considering the substantial impact that gastrointestinal symptoms can have on certain patients, such as limiting social activities due to the fear of experiencing episodes of faecal incontinence in public settings, the desirable anticipated effect of alleviating these symptoms is considered significant by the GDG. |  |
|  | Are the undesirable anticipated effects small? | ○ No  ○ Probably no  ○ Uncertain  ○ Probably yes  ● Yes  ○ Varies | No adverse effects of pelvic floor physiotherapy have been reported in the international literature available [3]. |  |
|  | Are the desirable effects large relative to undesirable effects? | ○ No  ○ Probably no  ○ Uncertain  ○ Probably yes  ● Yes  ○ Varies |  |  |
| Resource use | Are the required resources small? | ○ No  ○ Probably no  ○ Uncertain  ● Probably yes  ○ Yes  ○ Varies | The required resources only include the experienced healthcare professional (pelvic floor physiotherapist) to guide the patient through this process and to educate the patient. |  |
|  | Is the incremental cost small relative to the net benefits? | ○ No  ○ Probably no  ○ Uncertain  ● Probably yes  ○ Yes  ○ Varies | No evidence identified specific to this patient population. However, the GDG reckoned that the incremental costs (pelvic floor physiotherapy vs. no pelvic floor physiotherapy) are small relative to the potential benefits. |  |
| Equity | What would be the impact on health equity? | ○ Increased  ● Probably increased  ○ Uncertain  ○ Probably reduced  ○ Reduced  ○ Varies | No evidence identified specific to pelvic floor physiotherapy in this particular patient population. Considering the fact that no adverse events are reported after pelvic floor physiotherapy, the GDG reckons that the general health equity could be increased by this intervention. |  |
| Acceptability | Is the intervention acceptable to patients, their caregivers and healthcare providers? | ○ No  ○ Probably no  ○ Uncertain  ● Probably yes  ○ Yes  ○ Varies | The GDG considered the acceptability of this intervention for clinicians and policymakers. Patient acceptance depends on individual preferences concerning adherence to guided therapy/exercise sessions for symptom management. | The GDG reckons that this should only be incorporated as a second line treatment. |
| Feasibility | Is the intervention feasible to implement? | ○ No  ○ Probably no  ○ Uncertain  ● Probably yes  ○ Yes  ○ Varies | The feasibility of implementing this intervention may vary among hospitals or healthcare facilities and potentially across countries. Successful implementation of the intervention requires an experienced team of healthcare professionals. The availability may be affected by variations in funding between countries. | The GDG stated that this intervention is only feasible if there is an experienced therapist to guide the patients. |
| Overall ranking | | Conditional recommendation | | |

**5. Pelvic floor physiotherapy**

| ***Problem***: Gastrointestinal symptoms  ***Option***: Sacral Neuromodulation  ***Comparison***: No Sacral Neuromodulation  ***Setting***: In hospital, tertiary referral centre | | | ***Background and Objective***: Sacral neuromodulation is a well-established treatment option for patients with faecal incontinence. However, the eligibility criteria for this treatment limit its application to a relatively small number of patients, resulting in a limited worldwide experience. Considering that many patients are also experiencing faecal incontinence or low anterior resection syndrome after non-oncological colorectal resections, sacral neuromodulation could be a treatment option worth considering for this particular patient population. This question addresses whether sacral neuromodulation should be performed in patients experiencing faecal incontinence and/or other gastrointestinal symptoms after non-oncological colorectal resections. | |
| --- | --- | --- | --- | --- |
|  | **Criteria** | **Judgements** | **Research evidence** | **Additional considerations** |
| Problem | Is there a problem priority? | ○ No  ○ Probably no  ○ Uncertain  ○ Probably yes  ● Yes  ○ Varies | Benign colorectal resections are performed to address a spectrum of non-oncological conditions, including diverticulitis, inflammatory bowel disease (IBD), polyposis, functional bowel disorders or endometriosis. While the surgical procedure mirrors that of oncological cases, the primary distinction lies in the preservation of lymph nodes in these non-oncological resections [1]. Nevertheless, irrespective of the underlying condition, any type of colorectal resection could have an adverse impact on the patient’s bowel function [2-6]. Bowel dysfunction can manifest in a wide spectrum of symptoms, including urgency complaints, constipation, faecal incontinence and/or abdominal pain, all of which require very different management strategies [8, 9]. Understanding the range of gastrointestinal outcomes, their impact and consequences following non-oncological colorectal resections can improve quality of life in this cohort of patients. Therefore, the GDG, in collaboration with patient representatives, has identified the issue of gastrointestinal symptoms after colorectal surgery as a priority. | This evidence only applies to patients with a pouch. |
| Benefits & harms of the options | What is the overall certainty of this evidence? | ○ No included studies  ● Very low  ○ Low  ○ Moderate  ○ High | \| **No. of studies** \| **Design** \| **Limitations (RoB)** \| **Indirectness of patients, intervention and comparator** \| **Inconsistency** \| **Imprecision** \| **Other considerations** \| **Quality of evidence** \| \| --- \| --- \| --- \| --- \| --- \| --- \| --- \| --- \| \| 2 \| Cohort study \| Moderate \| Estimates of effect very different across studies \| None \| None \| None \| **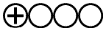** \| |  |
|  | Is there important uncertainty about how much people value the main outcomes? | ○ Yes  ○ Possibly  ● Probably not  ○ No  ○ Unknown | GDG members, including patient representatives, noted that there is no important variability or uncertainty in patients’ values and preferences. Specifically, patients would highly prioritise a reduction in the burden of disease associated with gastrointestinal symptoms after non-oncological colorectal resections. |  |
|  | Are the desirable anticipated effects large? | ○ No  ○ Probably no  ○ Uncertain  ● Probably yes  ○ Yes  ○ Varies | The expected positive effects are considerable, extending beyond mere alleviation of physical symptoms like the improvement of faecal incontinence or the reduction of defection episodes per day. The Quality of Life questionnaires have also demonstrated significant improvement in patients experiencing faecal incontinence after an oncological low anterior resection when employing this intervention [4]. This improvement has not yet been documented in this particular patient population after resections for benign indications. Nevertheless, similar results could be anticipated. |  |
|  | Are the undesirable anticipated effects small? | ○ No  ○ Probably no  ○ Uncertain  ● Probably yes  ○ Yes  ○ Varies | As with any surgical intervention, the occurrence of adverse events is possible. Reported complication rates range from 15-35% in international literature and include pain, infection, loss of efficacy and lead and/or pocket complications [5]. |  |
|  | Are the desirable effects large relative to undesirable effects? | ○ No  ○ Probably no  ○ Uncertain  ● Probably yes  ○ Yes  ○ Varies |  |  |
| Resource use | Are the required resources small? | ○ No  ● Probably no  ○ Uncertain  ○ Probably yes  ○ Yes  ○ Varies | No evidence identified specific to this patient population. The GDG considered costs and resource use when comparing SNM versus no SNM. A procedure in the operating theatre under sedation or even general anaesthesia is required for this intervention. | The GDG stated that funding is still lacking in many countries and/or hospitals. |
|  | Is the incremental cost small relative to the net benefits? | ○ No  ○ Probably no  ● Uncertain  ○ Probably yes  ○ Yes  ○ Varies | No evidence identified specific to this surgical patient population. A cost-effectiveness analysis has been performed on sacral neuromodulation for the treatment of faecal incontinence [6]. The authors conclude that SNM could be ‘an efficient investment with an acceptable incremental cost-effectiveness ratio’. |  |
| Equity | What would be the impact on health equity? | ○ Increased  ● Probably increased  ○ Uncertain  ○ Probably reduced  ○ Reduced  ○ Varies | No evidence identified specific to SNM on health equity in this particular patient population. Considering the fact that both physical and mental aspects are reported to improve after SNM, the GDG reckons that the general health equity could be increased by use of SNM. |  |
| Acceptability | Is the intervention acceptable to patients, their caregivers and healthcare providers? | ○ No  ○ Probably no  ○ Uncertain  ○ Probably yes  ○ Yes  ● Varies | The GDG deliberated on whether clinicians and policymakers would find this intervention an acceptable option. Patient acceptance of this intervention depends on individual preferences regarding the use of surgical interventions to manage their symptoms. | Funding for this intervention varies strongly between countries. |
| Feasibility | Is the intervention feasible to implement? | ○ No  ○ Probably no  ○ Uncertain  ● Probably yes  ○ Yes  ○ Varies | The feasibility of implementing this intervention may vary among hospitals or healthcare facilities and potentially across countries. Successful implementation of the intervention requires an experienced team of healthcare professionals. The availability may be affected by variations in funding between countries. | The GDG stated that SNM is not yet available everywhere in Europe. |
| Overall ranking | | Conditional recommendation | | |

**6. Sacral Neuromodulation**

**References**

1. Fujii S, Tsukamoto M, Fukushima Y, Shimada R, Okamoto K, Tsuchiya T, et al. Systematic review of laparoscopic vs open surgery for colorectal cancer in elderly patients. World J Gastrointest Oncol. 2016;8(7):573.

2. Dubernard G, Piketty M, Rouzier R, Houry S, Bazot M, Darai E. Quality of life after laparoscopic colorectal resection for endometriosis. Human Reproduction. 2006;21(5):1243-7.

3. Egger B, Peter MK, Candinas D. Persistent symptoms after elective sigmoid resection for diverticulitis. Dis Colon Rectum. 2008;51:1044-8.

4. Garavaglia E, Inversetti A, Ferrari S, De Nardi P, Candiani M. Are symptoms after a colorectal segmental resection in deep endometriosis really improved? The point of view of women before and after surgery. Journal of Psychosomatic Obstetrics & Gynecology. 2018;39(4):248-51.

5. Levack MM, Savitt LR, Berger DL, Shellito PC, Hodin RA, Rattner DW, et al. Sigmoidectomy syndrome? Patients' perspectives on the functional outcomes following surgery for diverticulitis. Dis Colon Rectum. 2012;55(1):10-7.

6. McGuire B, Brannigan A, O'Connell P. Ileal pouch–anal anastomosis. Journal of British Surgery. 2007;94(7):812-23.

7. Mege D, Meurette G, Vitton V, Leroi AM, Bridoux V, Zerbib P, et al. Sacral nerve stimulation can alleviate symptoms of bowel dysfunction after colorectal resections. Colorectal Dis. 2017;19(8):756-63.

8. Magdeburg J, Glatz N, Post S, Kienle P, Rickert A. Long‐term functional outcome of colonic resections: how much does faecal impairment influence quality of life? Colorectal Dis. 2016;18(11):O405-O13.

9. Wright HK. The functional consequences of colectomy. The American Journal of Surgery. 1975;130(5):532-4.

10. Fisher DA, Maple JT, Ben-Menachem T, Cash BD, Decker GA, Early DS, et al. Complications of colonoscopy. Gastrointestinal endoscopy. 2011;74(4):745-52.
